# STAG2 Expression is Associated with Adverse Survival Outcomes and Regulates Cell Phenotype in Muscle-invasive Bladder Cancer

**DOI:** 10.1158/2767-9764.CRC-22-0155

**Published:** 2022-10-06

**Authors:** Sarah R. Athans, Nithya Krishnan, Swathi Ramakrishnan, Eduardo Cortes Gomez, Sofía Lage-Vickers, Monika Rak, Zara I. Kazmierczak, Joyce Ellen Ohm, Kristopher Attwood, Jianmin Wang, Anna Woloszynska

**Affiliations:** 1 Department of Pharmacology and Therapeutics, Roswell Park Comprehensive Cancer Center, Buffalo, New York.; 2 Department of Bioinformatics and Biostatistics, Roswell Park Comprehensive Cancer Center, Buffalo, New York.; 3 University of Buenos Aires, Buenos Aires, C1053 CABA, Argentina.; 4 Department of Cell Biology, Jagiellonian University, 31-007, Krakow, Poland.; 5 Department of Cancer Genetics and Genomics, Roswell Park Comprehensive Cancer Center, Buffalo, New York.

## Abstract

Stromal antigen 2 (STAG2), in healthy somatic cells, functions in sister chromatid cohesion, DNA damage repair, and genome organization, but its role in muscle-invasive bladder cancer (MIBC) remains unknown. Here, using whole-exome and targeted sequencing (*n* = 119 bladder cancer clinical samples), we found several *STAG2* mutations in MIBC that correlate with loss of protein expression. The analysis of a bladder cancer tissue microarray (*n* = 346) revealed that decreased STAG2 protein expression is associated with improved overall and progression-free survival for patients with MIBC. In mouse xenograft studies, STAG2 knockdown (KD) decelerated MIBC tumor growth*,* whereas STAG2 overexpression accelerated tumor growth. In cell line studies, STAG2 loss augmented treatment with cisplatin, a first-line therapy for MIBC. STAG2 KD or overexpression did not alter degree of aneuploidy, copy-number variations, or cell-cycle distribution. However, unbiased RNA-sequencing analysis revealed that STAG2 KD altered gene expression. STAG2 KD led to significant downregulation of several gene sets, such as collagen containing extracellular matrix, external encapsulating structure organization, and regulation of chemotaxis. Therefore, we investigated the effect of STAG2 KD on cell migration and invasion *in vitro*. We found that STAG2 KD minimized cell speed, displacement, and invasion. Altogether, our results present a noncanonical function of STAG2 in promoting cell motility and invasion of MIBC cells. This work forms the basis for additional investigation into the role of STAG2 in transcriptional regulation and how it becomes dysregulated in *STAG2*-mutant MIBC.

**Significance:**

The cohesin component STAG2 regulates cell motility and invasion. STAG2 expression is associated with decreased MIBC survival and may be a useful biomarker to guide bladder cancer treatment.

## Introduction

Bladder cancer is the sixth most diagnosed cancer type in the United States, with an estimated 83,730 cases diagnosed in 2021 alone (1). It has one of the highest recurrence rates of all cancers with up to 74% of patients recurring within 10 years, and is four times more common in men than in women (2–6). Bladder cancer can present in two ways—non–muscle-invasive bladder cancer (NMIBC) and muscle-invasive bladder cancer (MIBC), depending on depth of invasion into the bladder wall. MIBC comprises up to 30% of new diagnoses and has a 5-year relative survival rate of only 47% (7, 8).

*Stromal antigen 2* (*STAG2*) is one of 12 genes significantly mutated in four or more cancer types, and is mutated in approximately 10% of bladder cancers (9). Most STAG2 mutations identified are truncating in nature, and lead to loss of STAG2 protein expression (10–13). STAG2 is a member of the multiprotein cohesin complex which is a ring-like structure that includes SCM1A, SMC3, and Rad21. During cell division, the cohesin complex encircles the sister chromatids, preventing premature separation until the onset of anaphase. At this point, Rad21 is proteolytically cleaved to allow the sister chromatids to separate properly into two daughter cells (14). Throughout this process, cohesin-STAG2 preferentially localizes to centromeres (15). As centromeric cohesin complexes are the last to be removed prior to anaphase, it was initially hypothesized that loss of STAG2 may cause errors in chromosome segregation, leading to aneuploidy and genome instability after cell division (16). Early studies in bladder cancer cell lines indicated that STAG2 loss was associated with alterations in modal chromosome number (12). However, these results have been inconsistent in subsequent studies, rendering the association controversial (11, 13).

In addition, the cohesin complex facilitates DNA replication fork progression (17), and cohesin-STAG2 specifically is recruited to sites of DNA double-stranded breaks in G_2_- and S-phase to facilitate repair by homologous recombination (18). Recent studies indicate that cohesin-STAG2 also contributes significantly to three-dimensional genome organization via maintenance of topologically associated domains, compartmentalization of chromatin, and contact between *cis*-regulatory elements of cell-type specific genes (19–23).

Clinically, STAG2 loss is associated with lower tumor grade and stage in patients with NMIBC and MIBC (11, 13, 21, 24). In addition, previous studies in MIBC have indicated that STAG2 status, that is, presence or loss, does not have an impact on overall (OS) or progression-free survival (PFS) outcomes (25). However, these studies do not quantify levels of STAG2 protein expression and its association to disease pathology or clinical outcomes in bladder cancer. Here, we interrogate a patient-derived tissue microarray (TMA) and find that the level of STAG2 protein expression varies broadly among patients. Interestingly, patients with MIBC with STAG2-low tumors have significantly improved clinical outcomes compared with STAG2-high tumor counterparts. These results emphasize the importance of quantifying STAG2 expression levels as opposed to classifying patients as “STAG2-presence” versus “STAG2-absence,” or “STAG2-wild type” versus “STAG2 mutant.” To identify functional changes that occur in MIBC when STAG2 protein expression is low, we utilize RNA sequencing (RNA-seq), *in vitro* assays measuring invasion, and xenograft growth rates *in vivo*. Our results form the basis for a new function of STAG2 in MIBC that may explain how STAG2 loss ultimately leads to better clinical outcomes.

## Materials and Methods

### Exome Sequencing

High-quality paired-end reads passing Illumina RTA filter were aligned to human reference genome (hg19) using Burrows-Wheeler Alignment (26). PCR duplicated reads are marked using Picard (http://picard.sourceforge.net/, RRID:SCR_006525). Somatic single-nucleotide variants and INDELs were called using neusomatic (27). Variants were manually reviewed and further filtered on the basis of: (i) the alternative allele is absent in the paired normal sample; (ii) Fisher exact test *P* value shows that number of reads with nonreference allele is significantly higher in tumor sample; (iii) mutant allele is present in both orientation; (iv) absent of photopolymers at variant position. Variants were annotated using ANNOVAR (28) with NCBI Ref Seq database to evaluate potential biological impact.

### IHC and TMA Analysis

Formalin-fixed paraffin-embedded sections were placed slides, deparaffinized, and incubated with STAG2 antibody (Cell Signaling Technology, catalog no. 5882, RRID:AB_10834529), Ki67 antibody (Abcam, catalog no. ab15580), or Cleaved Caspase 3 (Cell Signaling Technology, catalog no. 9664). Biotinylated anti rabbit (Vector BA-1000) was applied for 30 minutes followed by Elite ABC (Vector PK6100) for 30 minutes. DAB (diaminobenzidine; Dako; catalog no. K3468) was applied for 5 minutes for visualization. Slides were counterstained with hematoxylin then dehydrated, cleared, and cover slipped. TMA and IHC slides were digitally scanned using Aperio Scanscope (Aperio Technologies, Inc.) with 20× bright-field microscopy. These images were then accessible using Spectrum (Aperio Technologies, Inc.), a web-based digital pathology information management system. Aperio ImageScope version 11.2.0.780 (Aperio Technologies, Inc., RRID:SCR_014311) was used to view and analyze images. For additional details, see Supplementary Materials and Methods.

### Cell Culture

T24 cells (ATCC, catalog no. HTB-4, RRID:CVCL_0554) were cultured in McCoy's medium, UM-UC-3 (ATCC, catalog no. CRL-1749, RRID:CVCL_1783) in DMEM, TCC-SUP (ATCC, catalog no. HTB-5, RRID:CVCL_1738) in MEM plus 1x MEM non-essential amino acid, HB-CLS-1 (CLS, catalog no. 300190/p466_HB-CLS-1, RRID:CVCL_6213) in RPMI1640, BO2 in enriched F-12K medium supplemented with ROCK inhibitor Y-27632 (10 μmol/L) and insulin growth factor (5 μg/mL) as reported previously (29), SV-HUC (ATCC, catalog no. CRL-9520, RRID:CVCL_3798) in F12-K medium. Media for all lines was supplemented with 10% FBS and penicillin/streptomycin. All media purchased from Corning. All cell lines were maintained at 5% CO_2_, 37°C. Cells were tested for *Mycoplasma* via MycoAlert *Mycoplasma* Detection Kit (Lonza, catalog no. LT07-218) after thawing and minimally once every 3 months thereafter. All experiments were performed using cell lines passage 20 or below.

### Lentivirus-mediated Knockdown of STAG2

Two short hairpin RNAs (shRNA) targeting human *STAG2*, and a nontargeting control shRNA, were cloned into pGreenPuro shRNA Expression Lentivector (System Biosciences). The correct pGreenPuro shRNA constructs were verified by sequencing using H1 primer. The sequence of the shRNAs is as follows: shSTAG2-1: CCACTGATGTCTTACCGAAAT; shSTAG2-2: GCAAGCAGTCTTCAGGTTAAA; scrambled control: GCACTACCAGAGCTAACTCAGATAGTACT.

Human embryonic kidney cells, line 293TN (System Biosciences, catalog no. LV900A-1), were grown in 10 cm plates with DMEM (Invitrogen) containing 10% FBS and 0.1% penicillin-streptomycin. The cells were cultured to 90%–95% confluence and cotransfected with 2 μg of the shSTAG2/nontargeting control lentiviral constructs and 10 μg of the pPACKH1-plasmid mix (System Biosciences, catalog no. LV500A-1) using Lipofectamine 2000 (Invitrogen, catalog no. 11668-027). The viral supernatant was collected at both 48 and 72 hours after transfection and filtered using a 0.45 μm filter. Bladder cancer cells lines (T24, HB-CLS-1, TCC-SUP) were infected with lentivirus in the presence of 8 μg/mL of polybrene (Santa Cruz Biotechnology, catalog no. SC-134220). Transduced cells were enriched by puromycin selection for 1 week.

### STAG2 Overexpression

pEGFP (RRID: Addgene_165830) and FLAG tags were added to the 5′ end of the *STAG2* gene and amplified using PCR, then cloned into pCDH lentiviral vectors using 45 ng/μL pCDH and 30 ng/μL tagged *STAG2* (System Biosciences, catalog no. CD500). Cells were transformed following manufacturers’ instructions and plated on LB-agar plates. Colonies with identified insert were selected and grown overnight in LB media then screened by PCR. Construct was purified and transfected into HEK293T cells using pPACK Lentiviral Packaging Kit according to manufacturers’ instructions. Viral particles were collected after 48 hours and used to infect UM-UC-3 cells.

### Western Blot Analysis

Cells were cultured to 75%–80% confluence and lysed in Triton X-100/SDS lysis buffer (1% Triton X-100, 0.1% SDS, 50 mmol/L Tris, 150 mmol/L NaCl) containing protease inhibitors (Roche). Protein concentrations were determined using Bio-Rad protein assay (Bio-Rad, catalog no.500-0116). Equal amounts of protein lysates (50 μg) were resolved on a 4%–20% gradient SDS-PAGE and electrotransferred onto polyvinylidene difluoride membranes. Blots were incubated with STAG2 antibody (1:1,000, Cell Signaling Technology, catalog no. 5882, RRID: AB_10834529) overnight at 4°C. Horseradish peroxidase (HRP)-conjugated secondary antibodies were detected using Luminata Crescendo Western HRP Substrate (catalog no. WBLUR0100). The membranes were stripped using Restore Western Blot Stripping Buffer (Thermo Fisher Scientific, catalog no. 46430) and GAPDH antibody (1:2,000, Abcam, catalog no. ab9485, RRID: AB_307275) was used for assessment of protein loading.

### Animal Studies

Animal experiments were conducted and approved under our Institutional Animal Care and Use Committee protocol at Roswell Park. T24 (5*10^6^) and UM-UC-3 (1*10^6^) cells were subcutaneously injected bilaterally into the flank of nude mice. Only female mice were used to exclude sex as a biological variable. Control cells were injected on the left flank and experimental cells [STAG2 knockdown (KD) or overexpression] injected on the right. Tumor measurements were taken twice per week with calipers. Tumor volume was calculated with the following formula:



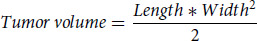



Mice were humanely euthanized when total tumor volume reached 2,000 mm^3^ or at days 56 and 14 for T24 and UM-UC-3 tumors, respectively.

### Mathematical Modeling of Tumor Growth Rates

The log tumor volume was modeled as a function of group, time, their interaction, and random subject effects using a linear mixed model. Tests about the appropriate contrasts of model estimates were used to compare tumor growth rates between groups. From the fitted models, estimates of the average time to reach 1,000 and 2,000 mm^3^ were obtained for each group.

### Clonogenic Assay

Cells were pretreated with 0.1 μg/mL (TCC-SUP, HB-CLS-1), 0.25 μg/mL (BO2) or 0.5 μg/mL (T24) cisplatin or control media for 24 hours. Treated cells were seeded in duplicate at 300 cells/well in a 6-well plate. After a 2-week incubation period at 37°C in a humidified atmosphere containing 5% CO_2_, clones were washed with PBS, fixed with 4% glutaraldehyde, and stained with 0.5% crystal violet. Clones were counted using a light microscope. Surviving fraction was calculated by dividing the cloning efficiency of treated cells by the cloning efficiency of untreated control cells.

### siRNA Transfections

Cells were transfected with 50 nmol/L of *STAG2* siRNA (SMARTpool siGENOME siRNA, Thermo Fisher Scientific) or control siRNA (On TargetPlus nonTargeting pool, Thermo Fisher Scientific) using Lipofectamine 2000 (Invitrogen). Forty-eight hours after, a second transfection was performed with same conditions as the first transfection. The cells were cultured for another 72 hours before harvesting for Western blot analysis.

### Somatic Copy-number Variation Analysis

Normalized data generated using GenomeStudio (RRID:SCR_010973) from Illumina were used for somatic copy-number variation (SCNV) and loss of heterozygosity (LOH) analysis. OncoSNP v1.4 (RRID:SCR_012985), a program to analyze SCNV and LOH events in cancer samples using SNP array data, was used to identify tentative SCNVs and LOHs. The automatically generated segmentation, copy number, and LOH results were reviewed manually using in-house R scripts to remove potential false-positive calls and identify missing events. Copy-number variation (CNV) analysis completed one time per cell line.

### Flow Cytometry

Cells were washed with PBS and fixed overnight in 70% ethanol at 4°C. For cell-cycle analysis, cells were stained with 50 μg/mL propidium iodide (Invitrogen, catalog no. P3566) and 0.2 mg/mL RNase (Thermo Fisher Scientific, catalog no. EN0531) for 1 hour at 4°C. Acquisition of data was performed using BD LSRII (BD Biosciences) and analyzed using ModFit software.

### RNA Isolation and cDNA Synthesis

Total RNA was extracted using the Direct-zol RNA MiniPrep kit (Zymo Research, catalog no. R2052) and quantified using a Nanodrop 8000 system (Thermo Fisher Scientific). cDNA was synthesized from 1 μg RNA using the iScript cDNA synthesis kit (Bio-Rad, catalog no.170-8891).

### RNA-seq and Gene Expression Analysis

Single-end raw sequencing reads were first preprocessed by using fastqc (v0.10.1; ref. 30) for sequencing base quality control. Reads were then mapped to RefSeq (RRID:SCR_003496) GRCh37-hg19 human reference genome and corresponding gene annotation obtained from UCSC's repository using splicing-detection tools Bowtie (v1.0.1, RRID:SCR_005476; ref. 31) and TopHat (v2.0.13, RRID:SCR_013035; ref. 32) allowing a maximum of one mismatch per read. A second pass QC was done using alignment output with RSeQC (v2.6.3, RRID:SCR_005275; ref. 33) to examine abundances of genomic features, splicing junction saturation, and gene-body coverage. Gene expression is quantified using HTSeq (34) using the -m intersection-strict option. Differential expression analyses were performed using DESeq2 (v1.18.1, RRID:SCR_000154; ref. 35), a variance-analysis package developed to infer and detect differential gene expression in RNA-seq data. Downstream and visualization plots were done using regularized log_2_ transformation implemented by DESeq2. Heatmaps were generated using the pheatmap (v1.0.8; ref. 36) R package. Enrichment analysis was performed using gene set enrichment analysis (GSEA; v4.1.0, RRID:SCR_005724), gene set c5.all.v7.5.symbols.gmt (Gene Ontology), with a minimum of 1,000 permutations performed per dataset. Permutations were based on gene set.

### Time-lapse Microscopy

Control, shSTAG2-1, and shSTAG2-2 T24 and HB-CLS-1 cells were plated at 60% confluence in 12-well plates. Twenty hours later, each plate was imaged for 30 hours (time-lapse 1 image per 5 minute) using a motorized-staged environment-controlled Leica AF6000 Live Cell Imaging System. Each condition was imaged in duplicate. Motility of cells was estimated by time-lapse monitoring of their trajectories. Tracks of individual cells were determined from the series of changes in cell centroid positions (30 hours with 5-minute intervals), pooled and analyzed as described previously (37).

### Transwell Migration and Invasion Assay

A total of 5 × 10^4^ cells (UM-UC-3) or 2.5 × 10^4^ cells (T24) in 500 μL of serum-free media were plated into each well of the upper chamber of a modified Boyden chamber (Corning BioCoat Matrigel Invasion Chamber, 8 μm pore size), and 750 μL of 10% FBS-enriched media was added to each bottom well. The polycarbonate membrane was either coated or uncoated with Matrigel matrix to simulate invasion and migration, respectively. After 24 hours incubation, the cells on the upper surface were scraped off, and the invasive cells attached to the lower surface of the membrane inserts were fixed and stained with crystal violet. The invading cells were observed and counted under a microscope in four random fields.

The percent invasion was calculated using the following formula:



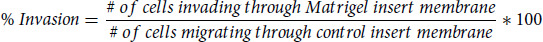



### Statistical Analysis

Quantitative measures are compared between groups using two-sample *t* tests. A *P* value of 0.05 was set to be statistically significant. The survival outcomes (OS and PFS) were summarized by STAG2 h-score (0–50 vs. 50–300) using standard Kaplan–Meier methods, with median and 1/3-year survival estimated with 95% confidence intervals. Comparisons were made using the log-rank test.

### Data Availability

Raw data for this study were generated at Roswell Park Comprehensive Cancer Center's Genomics Shared Resource. Derived data supporting the findings of this study are available from the corresponding author upon request.

## Results

### STAG2 is Clinically Relevant in MIBC

**FIGURE 1:**
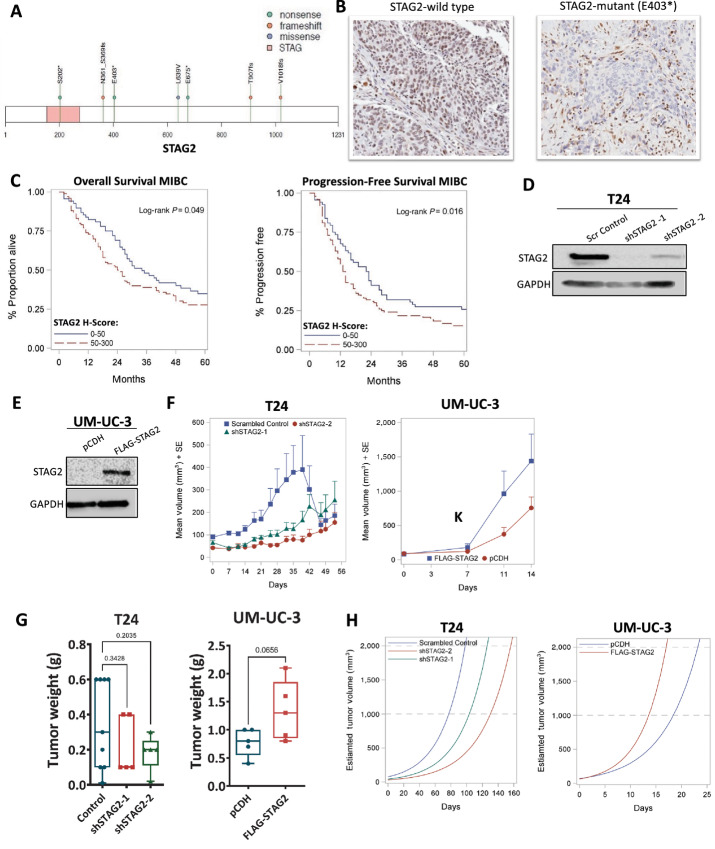
STAG2 is clinically relevant in MIBC. **A,** Graphical representation of the *STAG2* gene and mutations identified by sequencing of MIBC tumors (*n* = 119). STAG region: pink; nonsense, frameshift, and missense mutations shown in green, orange, and blue, respectively. **B,** Representative IHC staining for STAG2 in *STAG2*-WT MIBC tumor (left) or MIBC tumor with identified nonsense mutation (E403) in *STAG2* gene (right) **C.** Kaplan–Meier OS (left) and PFS (right) curves derived from MIBC TMA samples, stratified into STAG2-low (H-score 0–50, *n* = 69) and -high (H-score 50–300, *n* = 100) groups. *P* value computed using log-rank test; OS, *P* = 0.049; PFS *P* = 0.016. **D,** Western blot analysis showing STAG2 protein expression in T24 cells treated with scrambled control shRNA or two different shRNAs targeting STAG2 (shSTAG2-1, shSTAG2-2). GAPDH is used as loading control. Western blot analysis is representative of three independent experiments. **E,** Western blot analysis of UM-UC-3 cells transfected with control vector (pCDH) or FLAG-STAG2 to overexpress STAG2. GAPDH is used as loading control. Western blot analysis is representative of three independent experiments. **F,** Tumor volumes (mm^3^) for T24-scrambled control (*n* = 10), shSTAG2–1 (*n* = 5), and shSTAG2–2 (*n* = 5) tumors (left), or UM-UC-3 pCDH (vector control, *n* = 5) or FLAG-STAG2 (STAG2-overexpressed) tumors (right) bilaterally injected subcutaneously into the rear flank of nude mice. Data presented as mean + SEM. Volume calculated as defined in Materials and Methods. **G,** End of study tumor weights (g) from mice injected with T24 scrambled control, shSTAG2-1, and shSTAG2-2 cells (left) and UM-UC-3 pCDH (control) or FLAG-STAG2 (STAG2-overexpressed) cells (right). **H,** Mathematical modeling of estimated T24 (left) and UM-UC-3 (right) mouse xenograft tumor volume over time.

To investigate the prevalence of *STAG2* mutations, we performed whole-exome and targeted sequencing (*n* = 119) of tumor tissues from patients with MIBC. Sequencing revealed three nonsense mutations (S202, E403, and E675 at positions 123197899, 123189988, and 123179156, respectively), three frameshift mutations (N361_S369fs, T907fs, and V1018fs at positions 123211852, 123185034, and 123217398, respectively) and one missense mutation (L639V at 123197791) within the *STAG2* gene (Fig. 1A). To determine the impact of these mutations on STAG2 protein expression, we conducted IHC utilizing STAG2 antibody (Cell Signaling Technology mAb #5882) on the seven *STAG2*-mutant MIBC tissues in addition to 10 *STAG2*-wild type (WT) MIBC tissues. Tumors with nonsense or frameshift mutations were negative for nuclear STAG2 protein expression, but maintained stromal expression (Table 1; Fig. 1B, right). Conversely, the sample with missense mutation L639V and all but one (9/10) *STAG2*-WT sample were positive for STAG2 expression (Table 1; Fig. 1B, left). These results indicate that L639V missense mutation does not impact STAG2 expression, but nonsense and frameshift STAG2 mutations lead to loss of nuclear STAG2 protein in MIBC.

**TABLE 1:** Mutations and protein expression in bladder cancer patient samples

STAG2 Mutation	Tumor IHC Staining	Tumor source
No	Positive	Primary
No	Positive	Primary
No	Positive	Primary
No	Positive	Primary
No	Positive	Primary
No	Negative	Primary
No	Positive	Primary
No	Positive	Primary
No	Positive	Primary
No	Positive	Primary
Yes nonsense E403*	Negative (shown)	Primary
Yes nonsense E675*	Negative	Primary
Yes missense L639V	Positive	Primary
Yes frameshift T907fs	Negative	Primary
Yes nonsense S202*	Negative	Primary
Yes frameshift N361_S369fs	Negative	Primary
Yes frameshift V1018fs	Negative	Primary

To determine whether the level of STAG2 protein expression impacts patient outcomes in bladder cancer, we utilized a TMA comprised of 346 muscle-invasive and non–muscle-invasive bladder tumors. We detected STAG2 protein expression using IHC and sorted tumors into STAG2-low (H-score of 0–50) or STAG2-high groups (H-score 50–300; described in Materials and Methods). Clinical characteristics for each group are listed in Supplementary Table S1, and there were no significant differences within any single variable based on STAG2 expression. Next, we performed survival analyses. For patients with NMIBC, there was no difference in OS or PFS between STAG2-low and STAG2-high groups (Supplementary Fig. S1A). Interestingly, we found that in MIBC, patients with STAG2-low tumors had a median OS benefit of 9.5 months and significantly slower disease progression compared with STAG2-high patients (OS: 34.0 vs. 24.5 months, log-rank *P* = 0.049; PFS: 23.0 vs. 13.5 months, log-rank *P* = 0.016; Fig. 1C; Supplementary Table S2). Multivariate analysis for OS and PFS revealed a similar association for the cohort of patients with MIBC, with an adjusted HR of 1.43 for OS (*P* = 0.059) and 1.52 for PFS (*P* = 0.024; Supplementary Table S3). Although not statistically significant for OS, these results follow the same trend as our analysis based on STAG2 expression alone. Altogether, these data suggest that altered STAG2 levels in tumor tissues have different clinical consequences for patients with NMIBC and MIBC.

### STAG2 Loss Suppresses Tumor Growth *In Vivo*

Next, we analyzed the effect of STAG2 modulation on tumor growth *in vivo* using bladder cancer cell line–derived xenografts*.* Initially, we screened 18 different bladder cancer cell lines for STAG2 protein expression. STAG2 protein was detected in 17 of 18 lines, and UM-UC-3 was the only cell line with no STAG2 expression (Supplementary Fig. S1B). To determine the effect of STAG2 KD, we chose the well-characterized T24 MIBC cell line (38) which had one of the highest STAG2 protein expression levels. We transduced T24 cells with either control or two different shRNAs to generate stable KD of STAG2 (hereby referred to as shSTAG2-1 and shSTAG2-2; Fig. 1D). For STAG2 overexpression experiments, we utilized UM-UC-3 cells, also an MIBC-derived cell line, which have no STAG2 protein expression due to an endogenous truncating mutation (c.2946del; ref. 38). We infected STAG2-null UM-UC-3 cells with flag-tagged STAG2 or empty pCDH vector and confirmed STAG2 overexpression by Western blot analysis (Fig. 1E).

To assess the effect of STAG2 loss on tumor growth *in vivo*, we performed subcutaneous bilateral injections in nude mice, such that each mouse had one control tumor and one STAG2 KD tumor. Average volume of control tumors was consistently higher than tumors with shSTAG2-1 and shSTAG2-2 At week 5, two control tumors reached 2,000 mm^3^ and were humanely euthanized, thus lowering the average of the control tumors at all subsequent timepoints (Fig. 1F, left). Tumor weight at endpoint was greater in the control group compared with STAG2 KD tumors [0.345 g vs. 0.220 g (shSTAG2–1, *P* = 0.3428) and 0.184 g (shSTAG2-2, *P* = 0.2035)] (Fig. 1G left; Supplementary Fig. S1C). Because four STAG2 KD tumors were collected prior to endpoint because of the control tumor size from the bilateral injections, we used mathematical modeling to estimate tumor growth rates. Mathematical modeling allows for prediction of the average time each group would take to reach 1,000 and 2,000 mm^3^, which is otherwise beyond the scope of the experiment. The model predicted that control tumors would take 77.3 days to reach 1,000 mm^3^ and 98.2 days to reach 2,000 mm^3^, compared with 103.4 and 126.1 days for shSTAG2-1, and 130.3 and 156.1 days for shSTAG2-2 tumors for the same endpoints (Fig. 1H, left). Altogether, these results indicate that STAG2 loss slowed tumor growth which resulted in smaller endpoint tumor weights *in vivo.*

To analyze whether STAG2 overexpression may impact tumor progression *in vivo,* UM-UC-3 cells with empty pCDH vector or flag-tagged STAG2 were subcutaneously injected into nude mice. UM-UC-3 xenografts with STAG2 overexpression had larger tumor volumes throughout the duration of the experiment compared with tumors with empty vector (Fig. 1F, right). Endpoint tumor weights were higher in STAG2-overexpressing tumors compared with tumors with empty vector (1.34 g vs. 0.78 g, *P* = 0.0656; Fig. 1G, right; Supplementary Fig. S1C). Mathematical modeling of tumor growth rates predicted that STAG2-overexpressed tumors would reach 1,000 mm^3^ in 13.6 days and 2,000 mm^3^ in 17.0 days, faster than control tumors which were predicted to reach 1,000 mm^3^ in 18.4 days and 2,000 mm^3^ in 23.2 days (Fig. 1H, right). These results demonstrate that STAG2 overexpression contributes to faster growth rates and larger tumors *in vivo*.

Finally, we sought to directly assess whether STAG2 plays a role in proliferation *in vivo.* We utilized IHC analysis of Ki67 as a marker of proliferation in STAG2 KD tumors as well as STAG2-overexpressed tumors. Although our results did not reach statistical significance, most likely due to a modest sample size, T24 STAG2 KD tumors trended towards decreased proliferation (Supplementary Fig. S1D). We did not see a significant difference in Ki67 positivity between the UM-UC-3 control and STAG2-overexpressing tumors. Furthermore, we quantified level of apoptosis in each tumor via the expression of cleaved caspase 3 and TUNEL staining. We saw no significant differences in both our STAG2 KD and overexpression systems (Supplementary Fig. S1E and S1F). Together, our data from IHC analyses suggests that STAG2 does not play a role in tumor apoptosis, but may possibly influence tumor proliferation.

### STAG2 Loss Augments Cisplatin Treatment in MIBC

**FIGURE 2:**
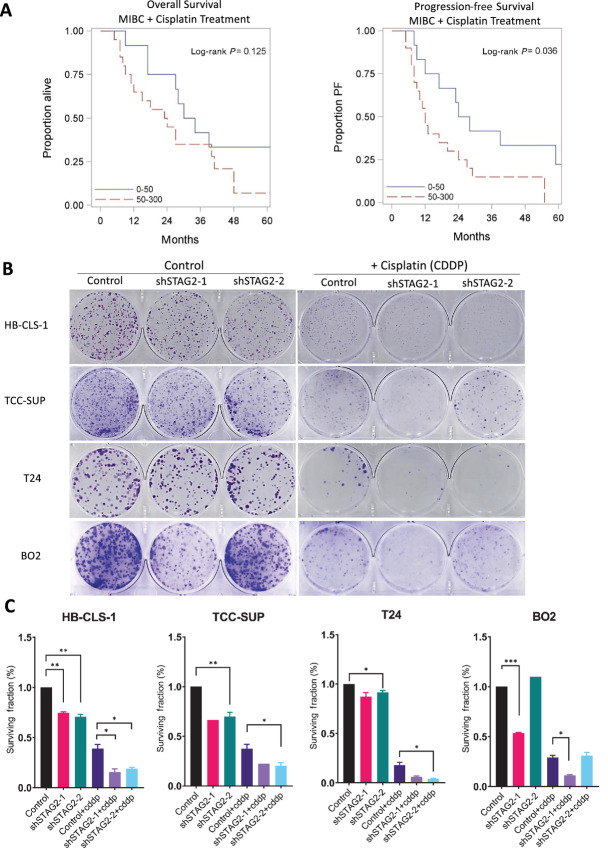
STAG2 loss augments cisplatin treatment in MIBC. **A,** Kaplan–Meier OS (left) and PFS (right) survival of MIBC TMA patient samples treated with cisplatin, stratified into STAG2-low (H-score 0–50, *n* = 12) and -high (H-score 50–300, *n* = 20) groups. *P* = 0.125 for OS, *P* = 0.036 for PFS; *P* value computed using log-rank test. **B,** Scanned images of crystal violet-stained HB-CLS-1, TCC-SUP, T24, and BO2 colonies after 24 hours of treatment and 14 days of growth. Left: control, shSTAG2-1, and shSTAG-2 clonal populations treated with vehicle. Right: populations treated with 0.1 μg/mL (TCC-SUP, HB-CLS-1), 0.25 μg/mL (BO2) or 0.5 μg/mL (T24) cisplatin. Images are representative of three individual experiments. **C,** Surviving fraction of cells relative to untreated control for HB-CLS-1, TCC-SUP, T24, and BO2 cell lines with and without cisplatin treatment. *P* values determined by unpaired Student *t* test. Comparisons made between untreated shSTAG2-1/2 and untreated controls, and shSTAG2-1/2+CDDP to control+CDDP. Data represented as mean + SEM. CDDP: cisplatin. Individual comparisons made using unpaired Student *t* test. *, *P* < 0.05; **, *P* < 0.01; ***, *P* < 0.001.

Cisplatin is frequently utilized as first-line therapy treatment for MIBC. Cisplatin treatment results in crosslinking of DNA bases that causes DNA damage and induces apoptosis. As part of the cohesin complex, STAG2 plays a role in DNA damage repair (18, 39). Therefore, we hypothesized that cells with STAG2 loss may have a deficiency in DNA damage response and thus be susceptible to damage induced by cisplatin. We first investigated our hypothesis in select patients with MIBC who received cisplatin treatment and analyzed the difference in OS and PFS of patients with STAG2-low versus STAG2-high tumors. Patients with low STAG2 tumor protein expression had a longer median OS compared with patients with high STAG2 tumor protein expression, although this difference did not reach statistical significance (32.0 vs. 23.5 months, *P* = 0.125). In addition, patients with low STAG2 tumor expression had significantly longer PFS compared with patients with high STAG2 tumor expression (26 vs. 12 months, *P* = 0.036; Fig. 2A; Supplementary Table S4). To confirm this observation, we designed *in vitro* experiments by treating cells with and without STAG2 expression*.* IC_50_ experiments with 48 hours of treatment in the T24 KD system revealed that STAG2 KD does not influence the sensitivity of MIBC cells to cisplatin in a short-term setting (Supplementary Fig. S2A; Supplementary Table S5). Therefore, we sought to investigate whether STAG2 KD impacts long-term growth after cisplatin treatment. For this purpose, we utilized T24 STAG2 KD cell lines and knocked down STAG2 in three additional MIBC cell lines (HB-CLS-1, TCC-SUP, and BO2). We confirmed STAG2 KD in isogenic cell lines by Western blotting (Supplementary Fig. S2B). We utilized a clinical-grade cisplatin formulation, and treated cells for 24 hours with vehicle (saline) or cisplatin (0.5 μg/mL for T24, 0.1 μg/mL for HB-CLS-1, TCC-SUP, and BO2) then seeded them at low density and analyzed clonogenic potential over 14 days. In all four MIBC cell lines, significant decrease in clonogenicity was observed in those cells with both STAG2 KD (shSTAG2-1 and shSTAG2-2) and cisplatin treatment compared with STAG2 KD or cisplatin alone (Fig. 2B and C). Our *in vitro* data and clinical observations support the conclusion that STAG2 loss augments the effect of cisplatin treatment in MIBC.

### Loss of STAG2 Does not Induce Aneuploidy or Alter Cell-cycle Distribution

**FIGURE 3:**
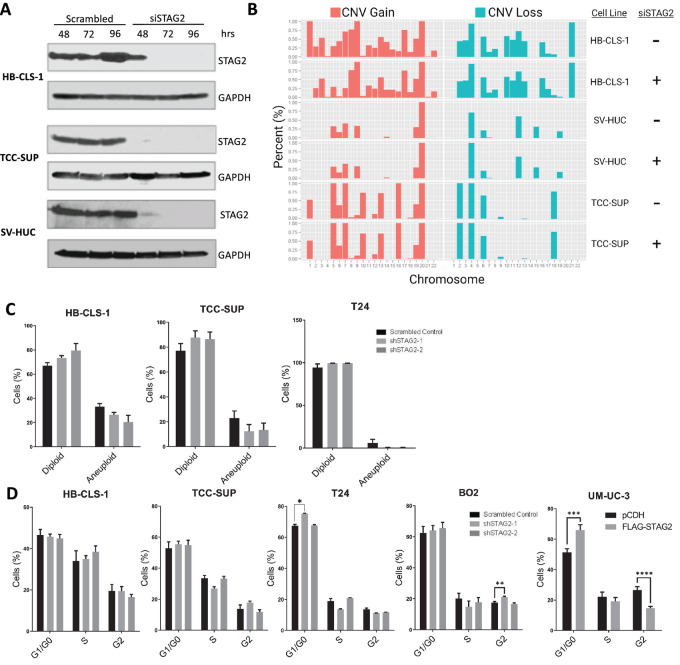
Loss of STAG2 does not induce aneuploidy or alter cell-cycle distribution. **A,** Western blot analysis for STAG2 in HB-CLS-1, TCC-SUP, and SV-HUC cells after treatment with 50 nmol/L scrambled control or STAG2 siRNA for 48, 72, or 96 hours. Figure representative of three independent experiments. **B,** CNV array indicating percentage CNV gained (red) or lost (blue) in each chromosome of HB-CLS-1, SV-HUC, or TCC-SUP cells treated with scrambled control or STAG2 siRNA for 96 hours. **C,** Percentage of diploid or aneuploid HB-CLS-1, TCC-SUP, or T24 cells after transfection with scrambled control, shSTAG2-1, or shSTAG2-2. Data from flow cytometry are represented as mean + SEM. **D,** Percentage of HB-CLS-1, TCC-SUP, T24, BO2, or UM-UC-3 cells in G_1_–G_0_-, S-, or G2-phase of the cell cycle in scrambled control, shSTAG2-1, or shSTAG2-2 (HB-CLS-1, TCC-SUP, T24, BO2), or pCDH or FLAG-STAG2 (UM-UC-3). Individual comparisons made using unpaired Student *t* test. Data from flow cytometry are represented as mean + SEM. *, *P* < 0.05; **, *P* < 0.01; ***, *P* < 0.001; ****, *P* < 0.0001.

STAG2 maintains sister chromatid cohesion (14); therefore, we investigated whether STAG2 loss would alter aneuploidy level in MIBC cells *in vitro*. We treated two MIBC cell lines (HB-CLS-1 and TCC-SUP) with siRNA against STAG2 or nonspecific siRNA for up to 96 hours to identify cellular alterations that occur after transient loss of STAG2. The immortalized normal bladder cell line SV-HUC was used as a control to determine basal level of aneuploidy in bladder cells. Western blotting indicated a complete KD of STAG2 in all three cell lines within 72–96 hours (Fig. 3A). SCNV analysis revealed that the tumor cell lines with nonspecific siRNA were already markedly aneuploid compared with normal SV-HUC cells. We did not observe a significant change in CNV gain or loss cell lines treated with STAG2 siRNA compared with their respective controls (Fig. 3B). To further determine changes in DNA content after long-term STAG2 KD, we performed flow cytometry using HB-CLS-1, TCC-SUP, and T24 shSTAG2-1 and shSTAG2-2 cell lines. Long-term STAG2 KD had no effect on the percentage of cells in diploid (defined as 2N-4N DNA content) compared with aneuploid (defined as greater than 4N DNA content) state in any cell line analyzed (Fig. 3C). Flow cytometry results were consistent with results from the SCNV array analysis. These results suggest that STAG2 does not alter aneuploidy in MIBC cells *in vitro*, and any effects seen after STAG2 KD in subsequent experiments are due to mechanisms not directly related to aneuploidy.

Because of its canonical role in cell division (40), we next investigated whether STAG2 loss can alter cell-cycle distribution in MIBC cell lines *in vitro*. We utilized four cell lines with STAG2 KD via shSTAG2-1/2 (HB-CLS-1, TCC-SUP, T24, and BO2) in addition to UM-UC-3 cells with STAG2 overexpression. Of all KD lines analyzed, T24 shSTAG2-1 cells showed a statistically significant increase of cells in G_1_–G_0_ phase, and BO2 shSTAG2-1 cells showed a significant increase of cells in G_2_-phase. Otherwise, STAG2 KD did not significantly alter the percentage of cells in each state of the cell cycle (Fig. 3D). Overexpression of STAG2 increased the percentage of cells in G_0_–G_1_ phase and decreased the percentage of cells in G_2_-phase in UM-UC-3 cells (Fig. 3D). Because there is no trend in cell-cycle changes across cell lines, these results suggest that STAG2 does not meaningfully affect cell-cycle distribution of MIBC cells.

### STAG2 KD Downregulates Extracellular Matrix and Migration Gene Sets

**FIGURE 4:**
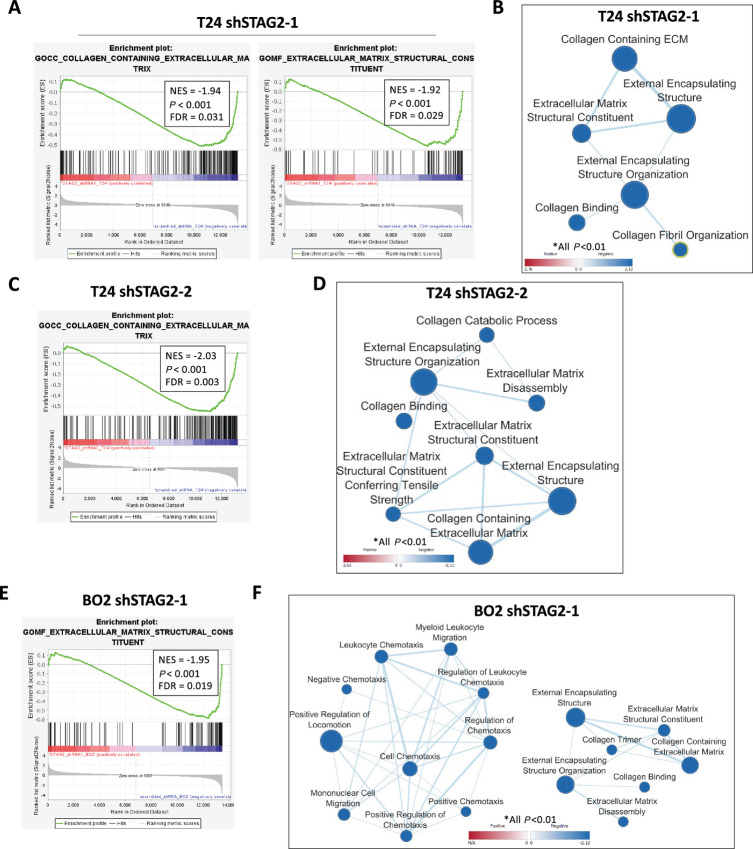
STAG2 KD downregulates ECM and migration gene sets. **A,** Representative gene set enrichment plot for gene sets differentially expressed between T24 shSTAG2-1 and control cells. **B,** Visualization of ECM-related gene sets significantly altered in shSTAG2-1 versus control T24 cells. Data visualized using Cytoscape software with EnrichmentMap plugin. Line thickness represents quantity of shared genes between gene sets, circle size represents number of genes significantly altered in each gene set, color represents degree of upregulation or downregulation (red: up, blue: down). Only gene sets *P* < 0.01 are shown. **C,** Representative gene set enrichment plot for shSTAG2-2 versus control T24 cells. **D,** Visualization of ECM-related gene sets significantly altered in shSTAG2-2 versus control T24 cells. Only gene sets *P* < 0.01 are shown. **E,** Representative gene set enrichment plot for BO2 shSTAG2-1 versus control cells. **F,** Visualization of ECM and migration related gene sets for BO2 shSTAG2-1 versus control cells. Only gene sets *P* < 0.01 are shown. FDR, false discovery rate; ECM, extracellular matrix.

Because we did not observe any chromosomal abnormalities related to the canonical functions of STAG2 in sister chromatid cohesion, sought to investigate alternative functions STAG2 in MIBC. We hypothesize that these noncanonical roles may contribute to the clinical outcomes related to levels of STAG2 protein expression in MIBC tumors. To identify these potential functions, we utilized an unbiased RNA-seq approach. We observed global gene expression changes after STAG2 KD in both T24 and BO2 cells. A total of 1,186, 3,762, and 452 genes were differentially expressed in T24 shSTAG2-1, T24 shSTAG2-2, and BO2 shSTAG2-1 cells, respectively, compared with matched controls treated with empty vector. To understand cellular processes that are over or underrepresented in STAG2 KD cells, we performed GSEA on these differentially expressed genes. Compared with controls, T24 shSTAG2-1 cells showed a significant downregulation of several gene sets related to the extracellular matrix (ECM), including collagen containing ECM (NES = −1.94, *P* < 0.001), ECM structural constituent (NES = −1.92, *P* < 0.001), and external encapsulating structure (NES = −1.8895, *P* < 0.01; Fig. 4A and B; Supplementary Fig. S3A). T24 shSTAG2-2 cells showed downregulation of similar gene sets, along with significant downregulation of gene sets encoding for ECM disassembly (NES = −1.814, *P* < 0.01) and collagen catabolic processes (NES = −1.821, *P* < 0.01; Fig. 4C and D; Supplementary Fig. S3B). BO2 shSTAG2-1 cells had a similar pattern of downregulated ECM gene sets. In addition, BO2 shSTAG2-1 cells had significant downregulation of gene sets related to cellular movement, including cell chemotaxis (NES = −1.831), mononuclear cell migration (NES = −1.726) and positive regulation of locomotion (NES = −1.793, all *P* < 0.01; Fig. 4E and F; Supplementary Fig. S3C). Altogether, RNA-seq results suggest that STAG2 plays a role in ECM structure and remodeling as well as movement in MIBC cells.

### STAG2 Increases Motility and Invasiveness of MIBC Cells

**FIGURE 5:**
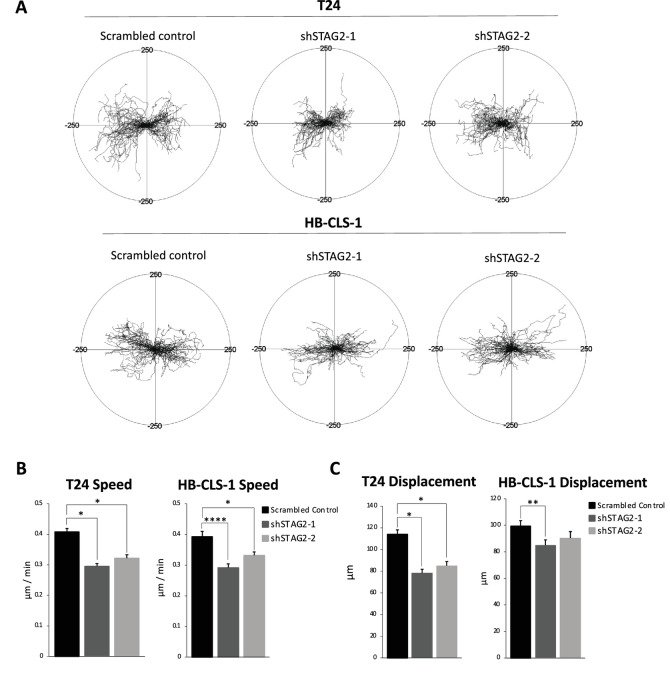
STAG2 increases motility characteristics of MIBC cells. **A,** Circular diagrams showing time-lapse microscopy monitoring of T24 and HB-CLS-1 control, shSTAG2-1, and sSTAG2-2 cell movement over 30 hours. Trajectories of single cells with the initial point of each trajectory placed at the origin of the plot (*n* = 100 cells/cell line/condition). Scale: −250 μm to 250 μm in *x* (horizontal) and *y* (vertical) directions. Average speed of cell movement (μm/minute; **B**) and displacement (μm; **C**) of T24 and HB-CLS-1 control, shSTAG2-1, and sSTAG2-2 cells over 30 hours. Data represented as mean + SEM. Individual comparisons made using unpaired Student *t* test. *, *P* < 0.05; ^**^, *P* < 0.01; ^***^, *P* < 0.001; ****, *P* < 0.0001.

RNA-seq analysis indicates that STAG2 may be involved in remodeling the ECM and cellular motility. Therefore, we investigated whether STAG2 loss alters bladder cancer cell movement *in vitro*. For this purpose, we performed time-lapse microscopy that shows single-cell resolution of cell speed and displacement, presented in the form of circular diagrams drawn with the initial point of each trajectory placed at the origin of the plot (Fig. 5A). T24 and HB-CLS-1 shSTAG2-1 and shSTAG2-2 cells moved significantly slower with shorter displacement over a 30-hour period compared to cells with empty vector (Fig. 5B and C). These data indicate that STAG2 expression is associated with an increase in cell movement.

**FIGURE 6:**
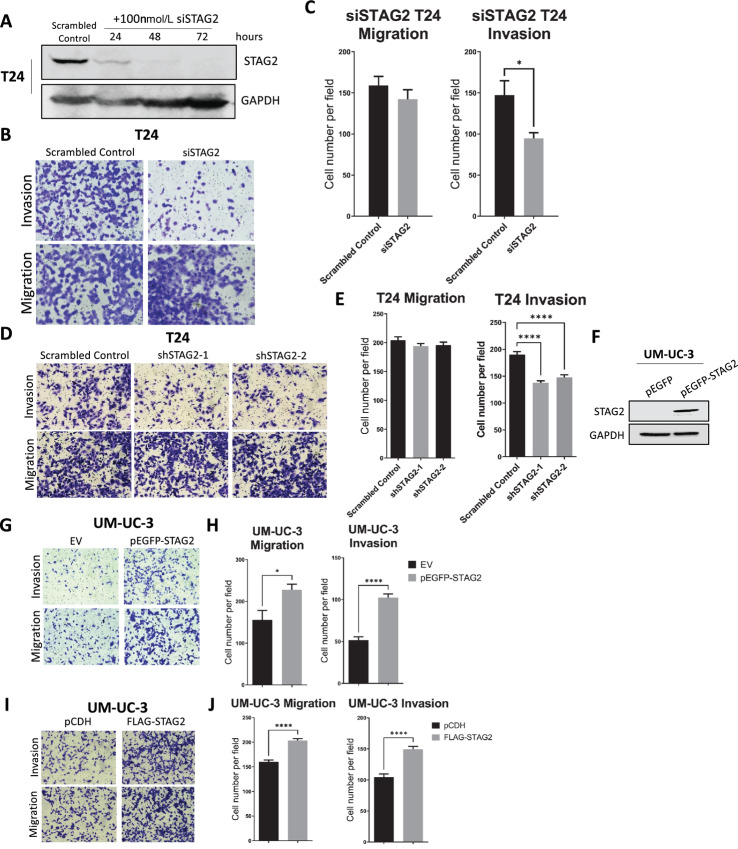
STAG2 increases invasiveness of MIBC cells. **A,** Western blot analysis demonstrating STAG2 protein expression in T24 cells transfected with scrambled control or STAG2 siRNA for 24, 48, and 72 hours. GAPDH used as loading control. Figure is representative of three independent experiments. **B,** T24 cells transfected with scrambled control or STAG2 siRNA, were seeded on top of a Transwell insert and allowed to travel through Matrigel-coated membrane (top, invasion) or uncoated membrane (bottom, migration) for 24 hours. **C,** Average number of T24 control or siSTAG2 cells per field view that migrated through uncoated membrane (left) or invaded through Matrigel-coated membrane (right) after 24 hours. **D,** T24 cells treated with scrambled control, shSTAG2-1, or shSTAG2-2, seeded in the top of a Transwell insert and allowed to move through Matrigel-coated membrane (top, invasion) or uncoated membrane (bottom, migration) for 24 hours. **E,** Average number of T24 control, shSTAG2-1, or shSTAG2-2 cells per field view that migrated through uncoated membrane (left) or invaded through Matrigel-coated membrane (right) after 24 hours. A minimum of 16 random fields were counted for each condition. **F,** Western blot analysis for STAG2 protein expression in UM-UC-3 cells transduced with pEGFP (control vector) or pEGFP-STAG2 (STAG2 overexpression vector). GAPDH used as a loading control. Figure is representative of three independent experiments. **G,** UM-UC-3 empty vector (EV) or pEGFP-STAG2 (STAG2-overexpressed) cells seeded in the top of a Transwell insert and allowed to move through Matrigel-coated membrane (top, invasion) or uncoated membrane (bottom, migration) for 24 hours. **H,** Average number of UM-UC-3 EV or pEGFP-STAG2 cells per field view that migrated through uncoated membrane (left) or invaded through Matrigel-coated membrane (right) after 24 hours. **I,** UM-UC-3 control (pCDH) or FLAG-STAG2 cells seeded in the top of a Transwell insert and allowed to move through Matrigel-coated membrane (top, invasion) or uncoated membrane (bottom, migration) for 24 hours. **J,** Average number of UM-UC-3 control or FLAG-STAG2 cells per field view that migrated through uncoated membrane (left) or invaded through Matrigel-coated membrane (right) after 24 hours. A minimum of 12 random fields were counted for each experiment. Data represented as mean + SEM. Individual comparisons made using unpaired Student *t* test. *, *P* < 0.05; ^**^, *P* < 0.01; ^***^, *P* < 0.001; ****, *P* < 0.0001.

ECM remodeling is a hallmark of epithelial–mesenchymal transition (EMT), and cells which undergo EMT are thought to be more invasive (41). Therefore, we determined whether STAG2 alters invasiveness of MIBC cells. To investigate short-term and long-term effects of STAG2 loss, we utilized T24 cells treated with siRNA and T24 shSTAG2-1/2 cells, respectively. siRNA effectively knocked down STAG2 within 72 hours in T24 cells (Fig. 6A). Cell migration and invasiveness was calculated via quantification of cells that traveled through Matrigel-coated and uncoated membranes, according to manufacturer's directions. T24 cells with siRNA against STAG2 and T24 shSTAG2-1/2 cells had no change in baseline migration through uncoated membranes. However, T24 cells with siRNA against STAG2 and T24 shSTAG2-1/2 cells were significantly less invasive than controls (Fig. 6B–E). Next, we utilized UM-UC-3 cells in which STAG2 was ectopically overexpressed (with FLAG or pEGFP tags; Figs. 1G and 6F). STAG2-overexpressing UM-UC-3 cells were significantly more invasive than control cells. In addition, STAG2 overexpression led to a significant increase in cell migration compared with control cells (Fig. 6G–J). Altogether, our results indicate that STAG2 expression in MIBC cells is associated with invasiveness.

## Discussion

*STAG2* is frequently mutated across several different cancer types, and here we show that it is frequently mutated in bladder cancer (9). Therefore, we sought to investigate the consequences of *STAG2* alterations for patients with bladder cancer. In this study, we show that patients with MIBC whose tumors have low STAG2 protein expression have improved OS and PFS, suggesting that the level of STAG2 expression may influence bladder cancer initiation or progression. STAG2 is frequently referred to as a tumor suppressor; however, our results and those of others indicate that STAG2 may drive oncogenic changes in bladder cancer (11, 12, 25). The role of STAG2 is likely cell-context dependent, which may explain why in some cancer types it has tumor-suppressive properties, and in others it has oncogenic properties (22, 23, 42, 43). Our results suggest that in the context of MIBC, STAG2 should not be classified as a tumor suppressor.

STAG2 protein level did not correlate with clinical outcomes for patients with NMIBC in our study, which is in contrast to results from a recent study (25). This study stratified patients as “STAG2-positive” and “STAG2 negative” (25). Tumors with low STAG2 expression may act similarly to “STAG2-negative” tumors but would have been classified as “STAG2-positive.” This different approach to classification may be a reason for observed discrepancy between previous reports and our study (25).

Here, we found that nonsense or frameshift *STAG2* mutations, but not missense mutations, are associated with loss of STAG2 protein expression. *STAG2* mutations, other than nonsense and frameshift, may lead to loss of tumor suppressor properties and a gain of tumor-promoting functions in bladder cancer. Further investigation of specific *STAG2* mutations is required to fully define these tumor-promoting functions. In addition, overexpressed STAG2 protein, which we observed in patients’ samples, may behave as *STAG2* gain-of-function mutations and contribute to STAG2 oncogenic properties. This may explain why we observed that higher STAG2 protein expression negatively influences patient outcomes in MIBC. Interestingly, one tumor that was *STAG2*-WT did not show any STAG2 protein expression. This may be due to hypermethylation of the *STAG2* promoter region leading to decreased expression of the STAG2 gene (10). This further emphasizes the importance of categorizing tumor samples by level of STAG2 protein expression instead of mutational status to account for epigenetic or post translational modifications which may ultimately affect expression. *In vivo,* we found that KD of STAG2 protein expression decreased tumor growth rate, while overexpression of STAG2 protein accelerated tumor growth rates and led to larger tumors. These results suggest that STAG2 expression can enhance tumor proliferation. Greater tumor proliferation may partially explain why patients with lower STAG2 expression experience better outcomes.

Cisplatin-mediated DNA cross-linking results in DNA damage, and STAG2 plays a role in DNA damage repair via homologous recombination (15, 17, 18, 42). This suggests that cells with STAG2 loss may not be able to successfully repair their DNA, resulting in susceptibility to DNA-damaging agents. This concept has been confirmed in glioblastoma, in which cells containing *STAG2* mutations show increased sensitivity to a combination of PARP inhibitors and DNA-damaging agents (44). Similarly, our *in vitro* results showed that STAG2 KD augmented the effects of cisplatin treatment in MIBC cells, leading to significantly decreased long-term viability compared with control cells treated with cisplatin. This could also explain the observations in the clinical data, where cisplatin-treated patients with low STAG2 expression had significantly longer PFS than their STAG2-high counterparts. Future studies to investigate STAG2 status as a predictive marker for response to cisplatin treatment in MIBC would help identify patients who would potentially benefit most from cisplatin, or other DNA-damaging therapies.

We investigated whether there was any impact on the STAG2’s canonical role in sister chromatid cohesion, of which results to date have been contradictory (13, 42, 45). We found that there were no significant changes in chromosomal CNV or in percentage of aneuploid cells after STAG2 KD. Because STAG2 is important for chromosome alignment prior to mitosis, we also investigated whether STAG2 loss caused errors in cell division by analyzing cell-cycle distribution. Our only significant findings were in the context of STAG2 overexpression in UM-UC-3 cells, in which we saw an increase of cells in G_1_-phase and a decrease of cells G_2_-phase of the cell cycle. This may be due to a G_2_-phase arrest in the context of mutant ATM, as ATM is important to the DNA damage response. Previous studies demonstrated that STAG2 plays an important role in DNA damage response (18, 39); therefore, in this context, the overexpression of STAG2 may compensate for the mutant ATM and allow more cells to progress through the cell cycle and reenter G_1_.

Overall, we saw no significant induction of aneuploidy or trends in cell-cycle distribution changes, suggesting that the canonical function of STAG2 in cell division is not affected after STAG2 KD. This may be because the STAG2 paralog, STAG1, can partially compensate for the loss of STAG2 to allow the continuation of the cell cycle and cell division (39, 40). Interestingly, we found that STAG2 KD greatly decreased cell speed, displacement, and invasion, revealing a noncanonical function of STAG2 in regulation of cellular movement. It is possible that STAG2–cohesin complexes may bind to regulatory regions of genes that promote migration and invasion, stimulating transcriptional activation and enhancing this invasive phenotype. Altogether, these results suggest that in MIBC, STAG2 has a separate function in addition to sister chromatid cohesion which is affected when STAG2 is lost.

STAG2 KD resulted in RNA expression changes of ECM related genes. These results suggest that STAG2 can regulate the transcriptome, indicating a role for STAG2 in gene expression separate from its role in sister chromatid cohesion. Currently, STAG2 is known to affect gene transcription as part of the cohesin complex. The cohesin–STAG2 complex colocalizes on DNA with CCCTC-binding factor (CTCF), forming DNA loops that alter transcription via activation, repression, or insulation (15). However, cohesin–STAG2 complexes frequently bind to sites that contain transcription factor motifs excluding the CTCF motif (46). These results suggest that STAG2 may work in concert with a separate group of transcription factors than previously thought to either induce or repress gene expression (46). It is worth noting that after loss of STAG2, these sites are not compensated for via cohesin–STAG1 complexes, indicating a STAG2-specific role in these areas of the genome. Importantly, many of these cohesin-STAG2–specific binding sites are in promoter and enhancer regions, pointing to a potential direct role of STAG2 in transcriptional regulation (20, 46). These sites are likely cell-type specific, potentially explaining why STAG2 may possess oncogenic properties in some cell contexts and act like a tumor suppressor in others. By altering transcription of invasion genes, STAG2 may encourage the invasive phenotype that we identified in the current study. Further investigation to identify STAG2-specific binding sites will be helpful to discern whether STAG2 directly or indirectly affects gene expression and determine the mechanism by which STAG2 loss alters cell behavior.

In conclusion, our study presents a novel function of STAG2 which leads to an aggressive cell phenotype. These data also form the basis for further investigation into the impact of STAG2 status in patients with MIBC and how to utilize this information for clinical applications.

## Supplementary Material

Supplementary Data S1 Supplementary methods, Supplementary Figures S1-S3, and Supplementary tables
