# Supplementary material for: STAG2 Expression is Associated with Adverse Survival Outcomes and Regulates Cell Phenotype in Muscle-invasive Bladder Cancer
Source: Cancer Res Commun. 2022 Oct 6;2(10):1129–43. doi: 10.1158/2767-9764.CRC-22-0155 (PMC9583756; doi:10.1158/2767-9764.CRC-22-0155)
Supplement: Supplementary Data S1 — Supplementary methods, Supplementary Figures S1-S3, and Supplementary tables [file crc-22-0155-s01.pdf]

Supplemental Information

Supplemental Methods

Immunohistochemistry and TMA analysis

Formalin-fixed paraffin sections were cut at 4 µm, placed on charged slides, and dried at 60°C for one hour. Slides were cooled to room temperature and deparaffinized through a series of xylenes and graded alcohols. Slides were pretreated in citrate buffer (Biocare CB910) for 60 minutes in a steamer then cooled for 20 mins. Slides were quenched in 0.3% H2O2 for 10 mins followed by protein block (Dako X0909) for 5 minutes. Slides were loaded onto the Dako Autostainer Plus and incubated with STAG2, Ki67, or cleaved caspase 3 for 60 minutes. Biotinylated anti rabbit (Vector BA-1000) was applied for 30 mins followed by Elite ABC (Vector PK6100) for 30 mins. DAB (Diaminobenzidine) (Dako; catalog #K3468) was applied for 5 minutes for visualization. Slides were counterstained with Hematoxylin then dehydrated, cleared, and cover slipped. TMA slides were digitally scanned using Aperio Scanscope (Aperio Technologies, Inc., Vista, CA) with 20x bright-field microscopy. These images were then accessible using Spectrum (Aperio Technologies, Inc., Vista, CA), a web-based digital pathology information management system. Aperio ImageScope version 11.2.0.780 (Aperio Technologies, Inc., Vista, CA) was used to view and analyze images. An annotation layer was created for each core of interest in the TMA targeting the cell of interest for image analysis. Regions were identified and annotated to represent the heterogeneity of staining of each TMA core and to reduce irrelevant regions from image analysis calculations. The Aperio platform was used to develop quantitative image analysis algorithm macros for the quantification of immunohistochemistry (IHC) slides. Briefly these algorithms used color de-convolution to separate diaminobenzidine (DAB) from the hematoxylin counterstain thereby providing stain separation. Each algorithm was tailored to fine tune the cell feature

detection using cellular, nuclear, and stain parameters, creating an algorithm macro based on the cell compartment location of the target protein. The macro was adjusted for the STAG2 antibody and bladder tissue to optimize results. The nuclear algorithm detects positive (DAB) nuclear staining for the individual tumor cells and quantifies their staining intensity. The analysis results provided the total number of detected cells, the percentage of cells per scoring class (0, 1+, 2+ and 3+) and the percentage of positive stained cells along with each sample's average staining intensity of the positive nuclei as a score of 0, 1+, 2+ and 3+. The H Score for each sample was calculated using the formula: (3 x percentage of strongly staining nuclei) + (2 x percentage of moderately staining nuclei) + (percentage of weakly staining nuclei), giving a range of 0 to 300.

Aperio ImageScope version 12.3.3.5048 was used to quantify IHC staining of Ki67 and cleaved caspase 3. Macro Positive Pixel Count v9 was utilized. Annotations representing tumor regions were created and the negative pen tool utilized to exclude background and staining artifacts. Each image was quantified via Positive Pixel Count v9, and positivity for each tumor was calculated according to the following formula:

$$Positivity = \frac{Total\ Number\ of\ Positive\ Pixels}{Total\ Number\ of\ Pixels\ (Positive + Negative)}$$

The following inputs were utilized for each tumor that was quantified:

Algorithm	Positive Pixel Count v9
Version	9.1
View Width	1000
View Height	1000
Overlap Size	0
Image Zoom	1.
Classifier	None
Class List	
Classifier Neighborhood	0
Pixel Area (millimeter-squared)	-1.
Hue Value (Center)	0.1
Hue Width	0.5

Color Saturation Threshold	4.e-002
Intensity Threshold WEAK (Upper Limit)	151
Intensity Threshold WEAK (Lower Limit)	175
Intensity Threshold MEDIUM (Upper Limit)	175
Intensity Threshold MEDIUM (Lower Limit)	100
Intensity Threshold STRONG (Upper Limit)	100
Intensity Threshold STRONG (Lower Limit)	0
Intensity Threshold Negative Pixels	-1

TUNEL Staining

TUNEL staining was performed using the TACS-XL In Situ Apoptosis Detection Kit – DAB (Cat #4828-30-DK) according to manufacturer's instructions. Quantification was performed as described above with the following algorithm specifications:

Algorithm	Positive Pixel Count v9
Version	9.1
View Width	1000
View Height	1000
Overlap Size	0
Image Zoom	1
Classifier	None
Class List	
Classifier Neighborhood	0
Pixel Area (millimeter-squared)	-1
Hue Value (Center)	0.1
Hue Width	0.5
Color Saturation Threshold	4.00E-02
Intensity Threshold WEAK (Upper Limit)	200
Intensity Threshold WEAK (Lower Limit)	150
Intensity Threshold MEDIUM (Upper Limit)	150
Intensity Threshold MEDIUM (Lower Limit)	125
Intensity Threshold STRONG (Upper Limit)	125
Intensity Threshold STRONG (Lower Limit)	0
Intensity Threshold Negative Pixels	-1

Cisplatin IC50 Assay

T24 scrambled control, shSTAG2-1, and shSTAG2-2 cells were seeded in triplicate wells of a 96 well plate at a density of 20,000 cells/well and treated with cisplatin at concentrations 16, 8, 4, 2,

1, 0.5, 0.25, 0.125, 0.0625, and 0 µg/mL for 48 hours. After 48 hours cells were fixed with 10% trichloroacetic acid for 1 hour, rinsed with water, stained with 0.057% sulfurhodamine B for 30 minutes, and rinsed with 1% acetic acid. Dye was solubilized with 10mM Tris base solution, pH 10.5, for 30 minutes then OD at 510 nm was measured using a microplate reader. Viability % was normalized to the average of the control wells (0 µg/mL) and graphed against concentration. IC50 values were calculated using GraphPad's non-linear regression analysis.

Supplemental Tables

Table S1. Clinical and demographic information for tissue microarray samples.

		0-50	> 50	Overall	P-value
Overall	N	137 (42.7)	184 (57.3)	330 (100%)	
Age	Mean/Std/N	68.77/11.32/137	68.48/11.30/184	68.61/11.29/321	0.928
	Median/Min/Max	70.00/36.00/91.00	69.50/39.00/88.00	70.00/36.00/91.00	
Gender	Male	106 (77.4%)	142 (77.2%)	256 (77.6%)	1
	Female	31 (22.6%)	42 (22.8%)	74 (22.4%)	
Race	White	134 (97.8%)	176 (95.7%)	319 (96.7%)	0.364
	Black	3 (2.2%)	8 (4.3%)	11 (3.3%)	
Smoking Hx	Never	28 (20.4%)	33 (17.9%)	62 (18.8%)	0.697
	Former	73 (53.3%)	92 (50.0%)	169 (51.2%)	
	Current	35 (25.5%)	58 (31.5%)	97 (29.4%)	
	Unknown	1 (0.7%)	1 (0.5%)	2 (0.6%)	
Alcohol Hx	No History	36 (26.3%)	50 (27.2%)	88 (26.7%)	0.829
	Past History	4 (2.9%)	9 (4.9%)	13 (3.9%)	
	Current Use	38 (27.7%)	48 (26.1%)	90 (27.3%)	
	Unknown Usage	59 (43.1%)	77 (41.8%)	139 (42.1%)	
Cancer Hx	No	104 (75.9%)	151 (82.1%)	261 (79.1%)	0.209
	Yes	33 (24.1%)	33 (17.9%)	69 (20.9%)	
Histology	Papillary Transitional Cell	64 (46.7%)	81 (44.0%)	147 (44.7%)	0.618
	Squamous Cell	4 (2.9%)	3 (1.6%)	7 (2.1%)	
	Transitional Cell	69 (50.4%)	100 (54.3%)	175 (53.2%)	
Grade	Grade I	9 (7.0%)	9 (5.2%)	19 (6.1%)	0.36
	Grade II	15 (11.6%)	31 (17.8%)	46 (14.7%)	
	Grade III	87 (67.4%)	105 (60.3%)	196 (62.8%)	
	Grade IV	18 (14.0%)	29 (16.7%)	51 (16.3%)	
Clinical AJCC	0	11 (25.0%)	13 (17.3%)	24 (19.4%)	0.249
	1-2	28 (63.6%)	45 (60.0%)	76 (61.3%)	
	3-4	5 (11.4%)	17 (22.7%)	24 (19.4%)	
Path AJCC	0	8 (10.5%)	14 (11.8%)	24 (11.9%)	0.844
	1-2	22 (28.9%)	38 (31.9%)	62 (30.7%)	
	3-4	46 (60.5%)	67 (56.3%)	116 (57.4%)	
Tx Delay	Mean/Std/N	0.12/0.44/137	0.15/0.52/184	0.13/0.48/321	0.594
	Median/Min/Max	0.00/0.00/3.00	0.00/0.00/3.00	0.00/0.00/3.00	
Intensity Score	Mean/Std/N	1.19/0.49/137	1.97/0.53/184	1.64/0.64/321	<.001
	Median/Min/Max	1.00/0.00/2.65	2.00/1.00/3.00	1.71/0.00/3.00	

Table S2. Overall and progression-free survival for non-muscle invasive and muscle invasive bladder cancer patients from tissue microarray analysis.

MIBC	Overall Survival		1-year Survival Rate (95% CI)	3-year Survival Rate (95% CI)	Median Survival (95% CI)	Median Follow-Up (Range)	Sample
		Total	0.78 (0.71, 0.84)	0.43 (0.36, 0.50)	28.0 (25.0, 37.0)	108.0 (2.0, 194.0)	E=134 C=35 T=169
		0-50	0.84 (0.73, 0.91)	0.48 (0.36, 0.59)	34.0 (27.0, 50.0)	137.0 (2.0, 194.0)	E=53 C=16 T=69
		50-300	0.74 (0.64, 0.81)	0.39 (0.29, 0.48)	24.5 (18.0, 30.0)	77 (30.0, 182.0)	E=81 C=19 T=100
	Progression-Free Survival		1-yr PFS Rate (95% CI)	3-yr PFS Rate (95% CI)	Median PFS (95% CI)	Sample	
		Total	0.64 (0.56, 0.70)	0.26 (0.20, 0.33)	16.0 (13.0, 20.0)	E=143 C=26 T=169	
		0-50	0.71 (0.58, 0.80)	0.32 (0.21, 0.43)	23.0 (15.0, 27.0)	E=56 C=13 T=69	
		50-300	0.58 (0.48, 0.67)	0.22 (0.14, 0.30)	13.5 (11.0, 17.0)	E=87 C=13 T=100	
NMIBC	Overall Survival		1-year Survival Rate (95% CI)	3-year Survival Rate (95% CI)	Median Survival (95% CI)	Median Follow-Up (Range)	Sample
		Total	0.95 (0.89, 0.97)	0.72 (0.64, 0.78)	94.0 (73.0, 111.0)	139.0 (11.0, 229.0)	E=89 C=56 T=145
		0-50	0.94 (0.84, 0.98)	0.72 (0.59, 0.81)	84.0 (48.0, 117.0)	119.0 (11.0, 172.0)	E=40 C=24 T=64
		50-300	0.95 (0.87, 0.98)	0.72 (0.61, 0.81)	101.0 (73.0, 126.0)	143.0 (18.0, 229.0)	E=49 C=32 T=81
	Progression-Free Survival		1-yr PFS Rate (95% CI)	3-yr PFS Rate (95% CI)	Median PFS (95% CI)	Sample	
		Total	0.75 (0.67, 0.81)	0.41 (0.33, 0.49)	26.0 (21.0, 35.0)	E=108 C=37 T=145	
		0-50	0.73 (0.61, 0.83)	0.38 (0.26, 0.50)	26.0 (17.0, 37.0)	E=47 C=17 T=64	
		50-300	0.75 (0.64, 0.83)	0.44 (0.32, 0.54)	29.0 (21.0, 50.0)	E=61 C=20 T=81	

Table S3. Multivariate analysis for overall and progression free survival of STAG2-high versus STAG2-low patients from tissue microarray analysis.

Cohort	Outcome	Adjusted HR (95% CI), p-value
MBIC	OS	1.43 (0.99, 2.07), p=0.059
	PFS	1.52 (1.06, 2.19), p=0.024

Table S4. Overall and progression-free survival for bladder cancer patients from tissue microarray analysis who were treated with cisplatin.

		1-year Survival Rate (95% CI)	3-year Survival Rate (95% CI)	Median Survival (95% CI)	Median Follow-Up (Range)	Sample
Overall Survival	Total	0.79 (0.61, 0.89)	0.36 (0.20, 0.52)	27.0 (17.0, 39.0)	59.0 (0.8, 59.0)	E=28 C=4 T=32
	0-50	0.92 (0.54, 0.99)	0.42 (0.15, 0.67)	32.0 (17.0, 87.0)	(58.0, 59.0)	E=10 C=2 T=12
	50-300	0.70 (0.45, 0.85)	0.35 (0.16, 0.55)	23.5 (9.0, 40.0)	(30.0, 31.0)	E=18 C=2 T=20
Progression-Free Survival		1-yr PFS Rate (95% CI)	3-yr PFS Rate (95% CI)	Median PFS (95% CI)	Sample	
	Total	0.67 (0.49, 0.80)	0.24 (0.21, 0.40)	17.0 (11.0, 25.0)	E=28 C=4 T=32	
	0-50	0.83 (0.48, 0.96)	0.42 (0.15, 0.67)	26.0 (9.0, 59.0)	E=10 C=2 T=12	
	50-300	0.55 (0.31, 0.73)	0.15 (0.04, 0.33)	12.0 (8.0, 24.0)	E=18 C=2 T=20	

Table S5. IC50 values for T24 control, shSTAG2-1 and shSTAG2-2 cells treated with clinical-grade cisplatin for 48 hours at concentrations of 16, 8, 4, 2, 1, 0.5, 0.21, 0.125, 0.625 and 0 µg/mL.

**IC50 Values**		**95% CI**
Control	0.9285	0.7884-1.094
shSTAG2-1	0.8506	0.7065-1.025
shSTAG2-2	1.079	0.8044-1.452

Supplementary Figure 1

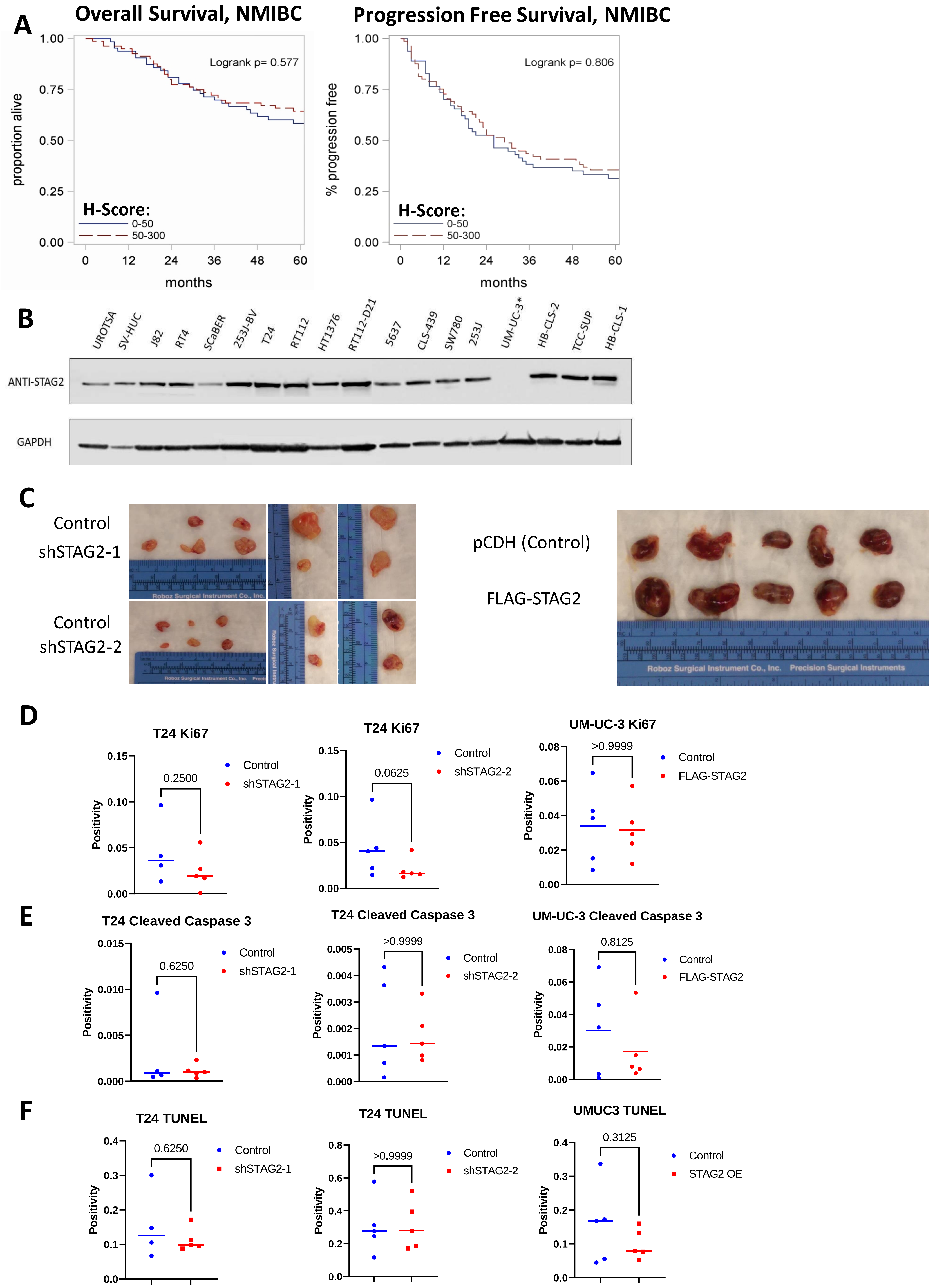

Figure S1.

A. Kaplan-Meier overall survival (left) and progression free survival (right) curves derived from NMIBC TMA samples, stratified into STAG2-low (H-score 0-50, n=64) and -high (H-score 50-300, n=81) groups. P-value computed using log rank test **B.** Western blot for STAG2 protein expression in 18 bladder cancer cell lines. GAPDH used as loading control. Figure is representative of three independent experiments. Asterisk (*) denotes UM-UC-3 line as the only line with absent expression. **C.** Images of bilateral tumors from each mouse at endpoint. Left, control tumors (n=9), shSTAG2-1 tumors (n=5) and shSTAG2-2 tumors (n=5). Right, pCDH (control) tumors (n=5) and FLAG-STAG2 tumors (STAG2 overexpression, n=5). **D.** Quantification of IHC staining for Ki67, a marker of proliferation, in mouse tumors shown in C. Positivity calculated as the number of positively stained pixels per tumor divided by the total pixel number of each tumor. **E.** Quantification of IHC staining for cleaved caspase 3, a marker of apoptosis, in mouse tumors shown in C. Positivity calculated as described in methods. P-values for IHC staining calculated using Wilcoxin matched-pairs signed rank test. **F.** Quantification of staining for TUNEL, a marker of apoptosis, in mouse tumors shown in C. Positivity calculated as described in methods. P-values for IHC staining calculated using Wilcoxin matched-pairs signed rank test.

Supplementary Figure 2

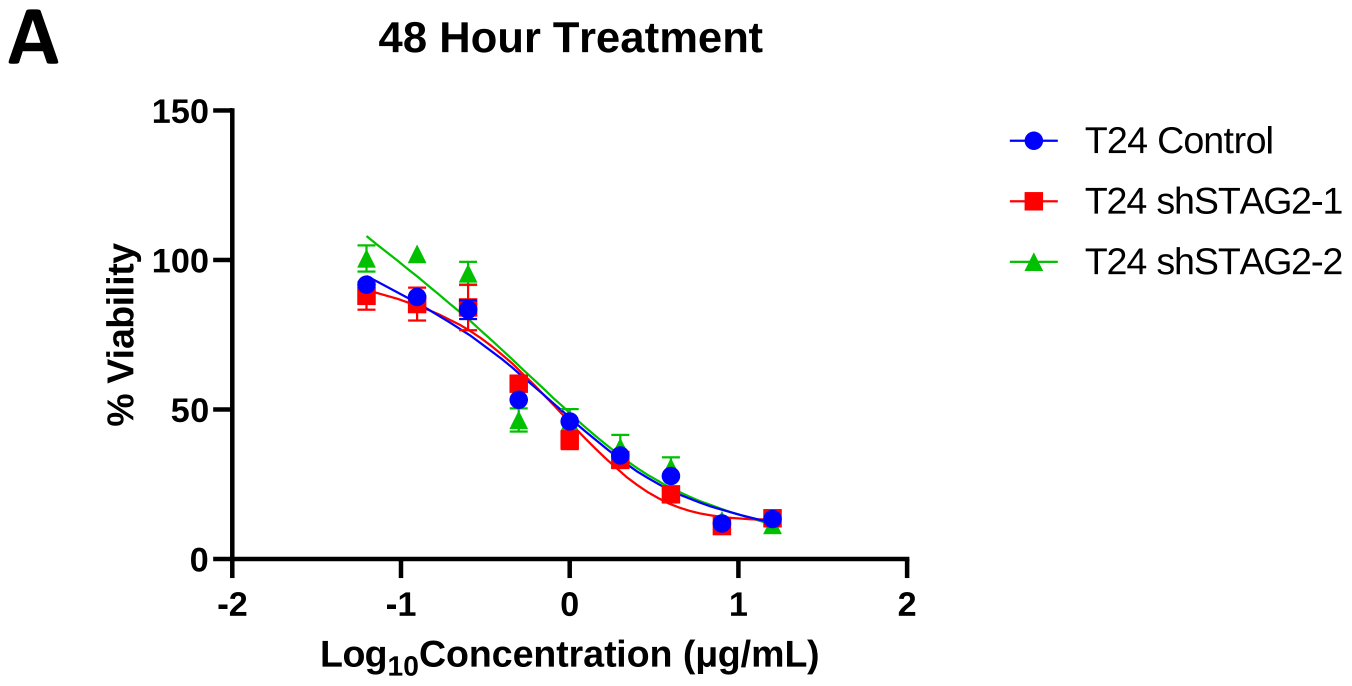

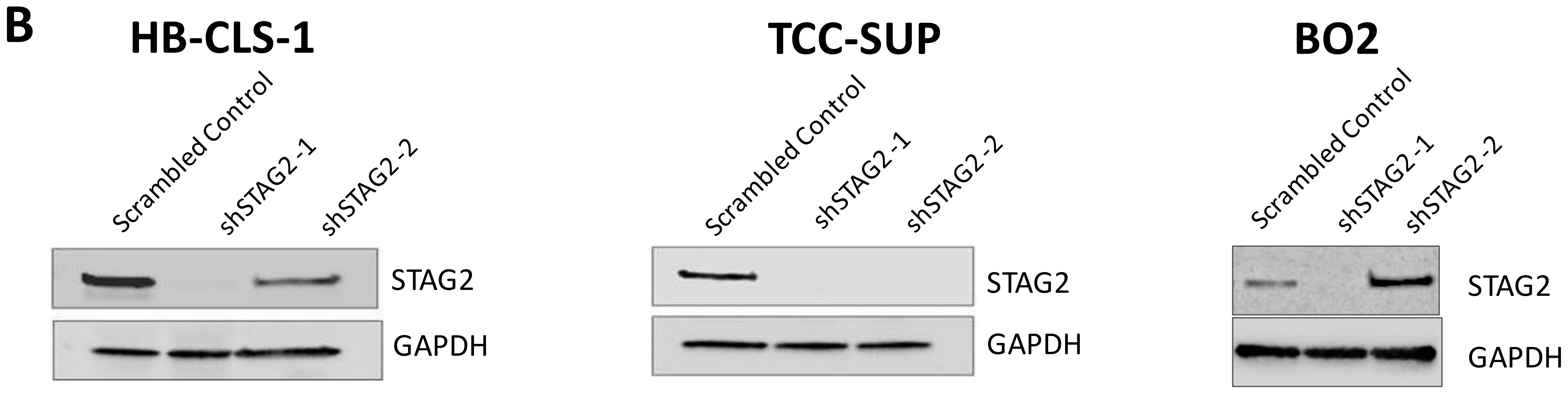

Figure S2.

A. IC50 curves for T24 scrambled control, shSTAG2-1 and shSTAG2-2 cells treated with increasing concentrations of clinical-grade cisplatin for a time period of 48 hours. Tabular IC50 values can be found in Supplementary Table 5. All concentrations were tested in triplicate. **B.** Western blots for STAG2 in HB-CLS-1 (right panel) TCC-SUP (middle panel) and BO2 (right panel) control, shSTAG2-1, or shSTAG2-2 cells. GAPDH used as loading control. Figures are representative of three independent experiments.

Supplementary Figure 3

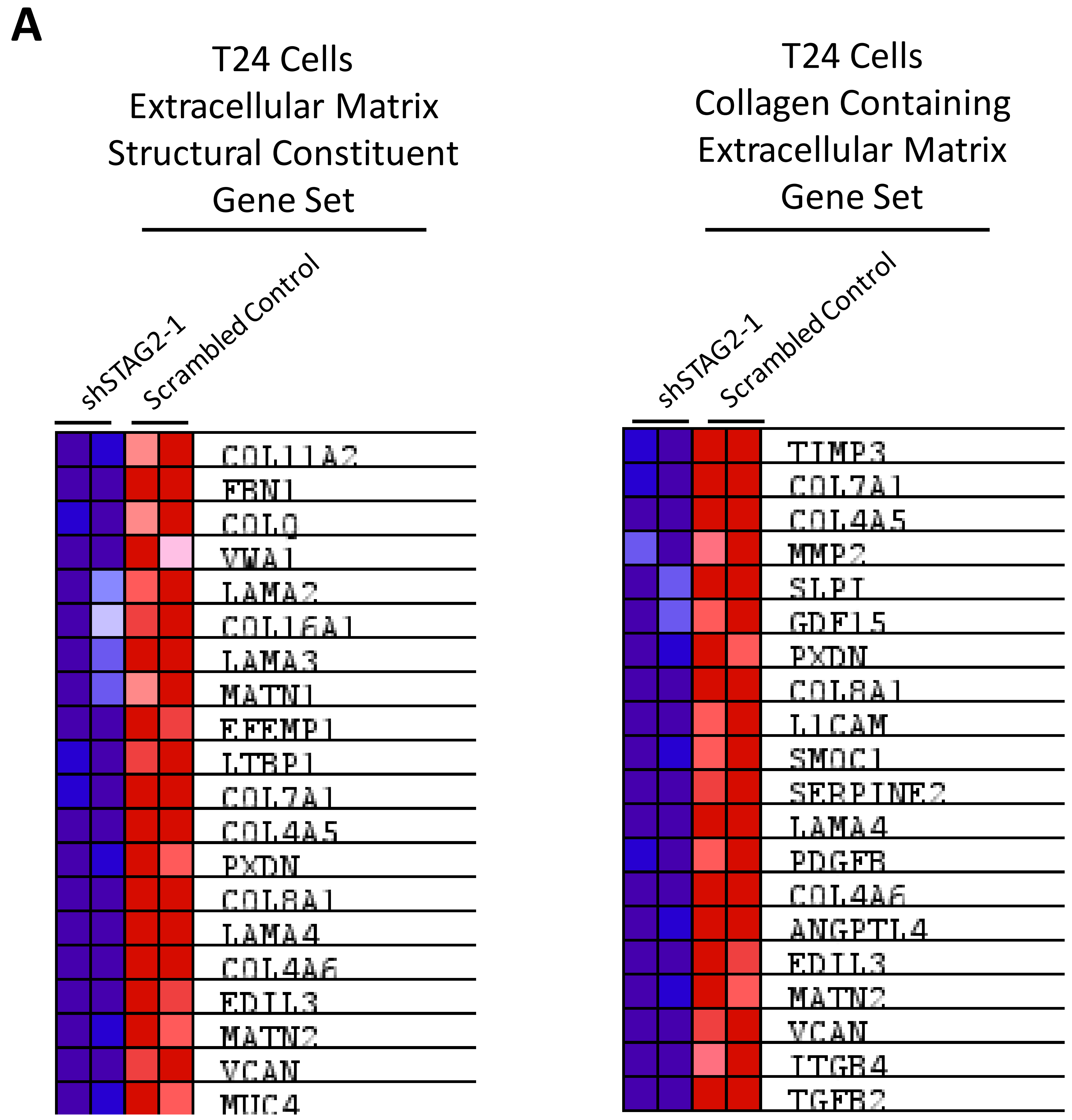

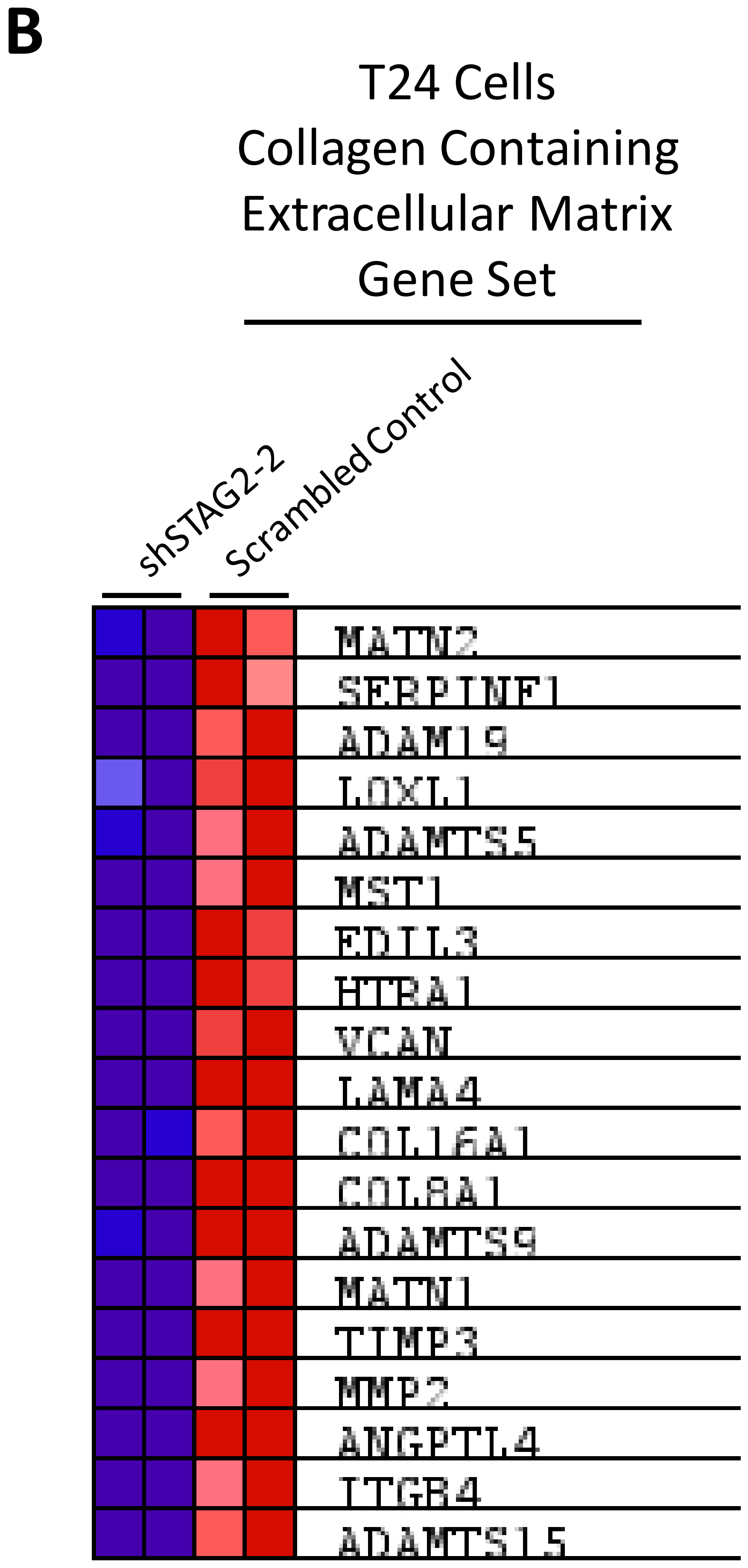

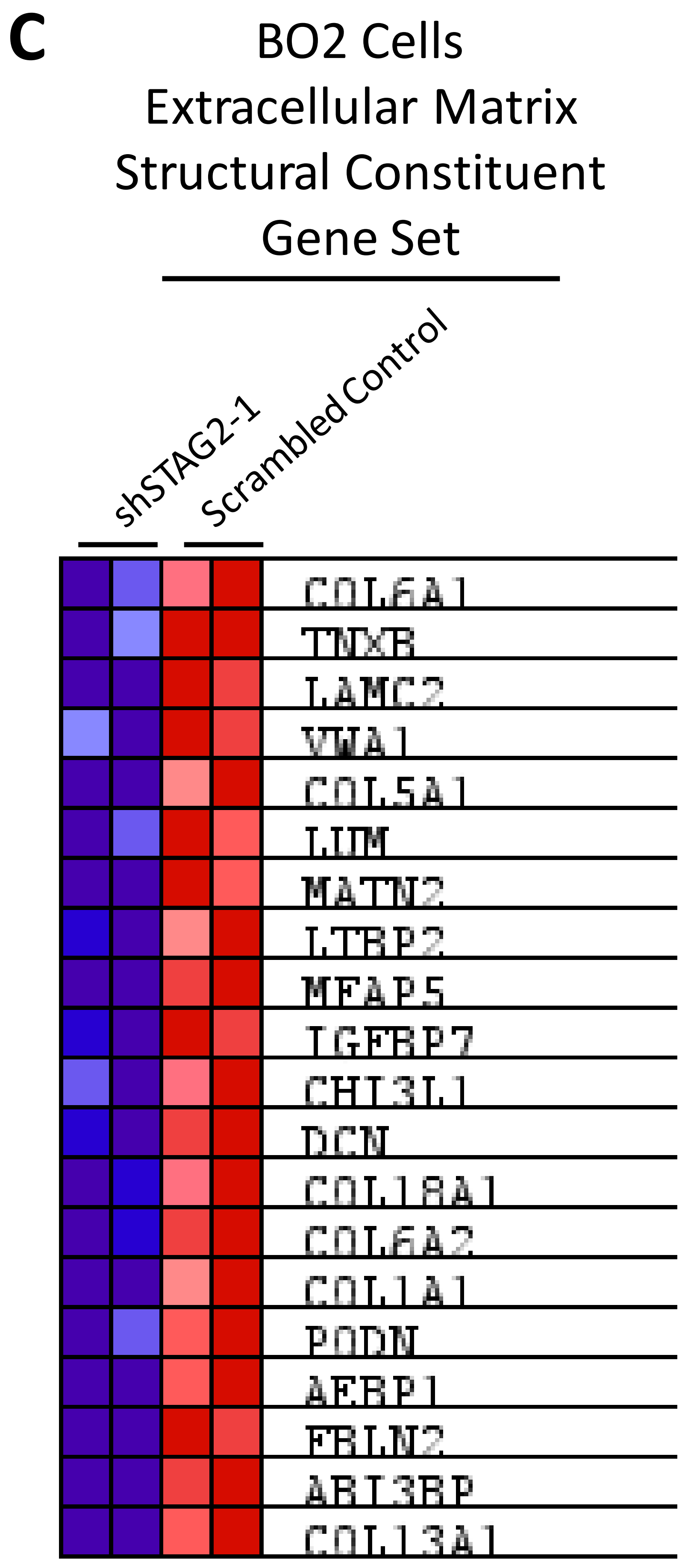

Figure S3.

A. Top 20 most downregulated genes in T24 shSTAG2-1 cells compared to controls in the gene set 'Extracellular Matrix Structural Constituent' (left) and 'Collagen Containing Extracellular Matrix' (right). **B.** Top 20 most downregulated genes in T24 shSTAG2-2 cells compared to controls in the gene set 'Collagen Containing Extracellular Matrix.' **C.** Top 20 downregulated genes in BO2 shSTAG2-1 cells compared to controls in the gene set 'Extracellular Matrix Structural Constituent.' Heatmaps generated using GSEA software. Red: upregulation of expression; blue: downregulation of expression.
